# Feasibility and acceptability of saliva-based testing for the screening of SARS-CoV-2 infection in prison

**DOI:** 10.3389/fpubh.2022.808030

**Published:** 2022-08-11

**Authors:** Chiara Parodi, Emerenziana Ottaviano, Nicola Cocco, Silvia Ancona, Silvia Bianchi, Valentina Massa, Raffaella Bartolotti, Barbara Pezzoni, Ruggero Giuliani, Elisa Borghi, Roberto Ranieri

**Affiliations:** ^1^Department of Health Sciences, Università degli Studi di Milano, Milan, Italy; ^2^Infectious Diseases Service, Penitentiary Health System, ASST-Santi Paolo e Carlo, Milan, Italy; ^3^San Vittore Health Unit, Penitentiary Health System, ASST-Santi Paolo e Carlo, Milan, Italy

**Keywords:** SARS-CoV-2, pandemic, prison, public health, saliva testing

## Abstract

**Background:**

Saliva molecular tests have shown a similar sensitivity and specificity compared to nasopharyngeal test for SARS-CoV-2 diagnosis in both symptomatic and asymptomatic individuals. The SARS-CoV-2 pandemic affected Lombardy prisons, generating the need for extensive contact tracing activities and for detecting asymptomatic carriers. The availability of a less invasive test in a setting that hosts a high-risk and often hard-to-reach population, suggests its possible use in prisons.

**Methods:**

The study was carried out on a population of new incomers in Milan San Vittore pre-trial prison. All the new incomers were submitted to quarantine and to saliva test and nasopharyngeal swab (NPS) for SARS-CoV-2 detection at the entry and at the end of quarantine before their admission in community (Protocol 1–February 2^nd^ to March 5^th^, 2021). Starting from March 8^th^ to July 30^th^, 2021, the screening protocol was adjusted to avoid biases in sample collection (Protocol II), and saliva testing was performed at entrance.

**Results:**

12/1,120 enrolled subjects were excluded from the study. Among the 1,080 processed samples, 1 tested positive, 5 weakly positive, 1,069 negative, 3 were invalid, and 2 samples tested positive for the viral gene N2 only, with Ct value above 38. During Protocol I, 6/156 coupled saliva/NPS tests were discordant due to food ingestion prior saliva collection, prompting us to establishing Protocol II.

**Conclusions:**

Saliva molecular testing is feasible in prison setting, being less invasive and easier to use, and reliable. Acceptability was very high even in a complex context as that of newly incarcerated persons.

## Introduction

More than 10 million people are incarcerated throughout the world (1). Due to their status of forced confinement and shared life, People in Prison (PiP) are more vulnerable to diseases than the general population: data show that prisons can serve as a source, amplification, and spread of infectious diseases inside and outside the prison setting (2). Health care disparities between PiP and the general population are common. This evidence has been ascribed to various factors, both concerning PiP's behavioral and socio-economic factors (such as smoking, alcohol misuse and intravenous drug abuse, and a subsequent increased risk of infectious diseases), and the structural deficiencies of prison health care in many places (3). Often PiP come from the social and poorer parts of the population, and they usually have a lower level of education, less access to care and consequently worse level of prevention and medical care (4).

The disproportionate burden of physical and psychiatric illness in PiP is a challenge from a public health perspective. In this context, for some of these individuals, prison offers an opportunity for diagnosis and treatment of illness that they would not receive in the community.

The response to COVID-19 in prisons and similar contest of detention is particularly challenging and requires a government and society-wide approach. Surveillance through molecular testing allows to avoid outbreaks within the prison and safeguard the health of the entire community that is part of it.

The SARS-CoV-2 pandemic deeply affected the Italian Region Lombardy and its local prisons, especially in the second wave (October 2020–March 2021) and this phenomenon reinforced the need for planned contact tracing activities and for detecting asymptomatic carriers (5). Since September 2020, antigenic nasopharyngeal swab for SARS-CoV-2 were performed to new incomers in Milano San Vittore pre-trial prison. Shortly thereafter, in a first phase, nasopharyngeal molecular testing was performed as a more sensible tool, and a 14-day quarantine was applied.

Saliva molecular tests have shown a similar sensitivity and specificity compared to nasopharyngeal tests in the diagnosis of SARS-CoV-2 in both symptomatic and asymptomatic patients (6, 7).

In this work we aim to demonstrate how self-administered molecular salivary testing is a viable choice over nasopharyngeal swabbing in our target community and in comparable realities.

## Materials and methods

### Study population and sample collection

We enrolled people who are kept in police custody in Milan San Vittore pre-trial prison, accommodating ~1,000 people daily and has an annual turnover of up to 5,000. Since the beginning of the COVID-19 outbreak, all new arrivals have been quarantined and nasopharyngeal swabbed for SARS-CoV-2 at the entry and at the end of quarantine before admission to the community. According to the procedures agreed with the Italian Ministry of Justice, PiP refusing SARS-CoV-2 testing had to stay in quarantine for 21 days.

We started the saliva protocol on February 2^nd^, 2021. Both salivary and nasopharyngeal molecular tests were performed together on the first working day (between day 2 and day 6) after the custody (Protocol I). On March 8^th^, 2021, to reduce biases in saliva collection, the protocol was revised as the molecular saliva test was performed at the jail admission (day 0), when subjects are in a fasting condition, and the NPS test was performed at the end of the quarantine (14 days) before entering the prison community (Protocol II). The enrolled period ended on July 30^th^, 2021.

The nasopharyngeal swabs (NPS) were performed using UTM Copan device (Copan, Italy), as a gold standard collection method for respiratory tract infections detection. The NPS is preserved in a virus transport medium (UTM) until processing and must be stored at 4°C. Saliva samples were collected using LolliSponge™ device (Copan, Italy), held for almost 1 min in the mouth. Once properly soaked in saliva, the device was stored at room temperature (RT) until used—up to 3 days—being the saliva self-preserving, without a transport medium.

### Nucleic acid extraction

RNA extraction from saliva and NPS was performed using the commercial QIAamp^®^ Viral RNA Mini Kit (Qiagen), following manufacturer's instructions. Briefly, the extraction process consists of five main steps: incubation in a Buffer + Carrier RNA solution; sample purification using ethanol (96–100%); and two further purifications using two different wash buffers in order to obtain pure RNA. The purified viral RNA was eluted using AVE buffer (Rnase-free water containing 0.04% sodium azide that prevents bacterial growth and therefore contamination of the sample).

### SARS-CoV-2 realtime qPCR

After extraction procedure, 5 μl of extracted RNA were used for RT-qPCR to the simultaneous detection of N1 (FAM probe), N2 (SUN) and human RNAse P genes (RP, ATTO647) using the protocol published by the Centers for Disease Control (8, 9). The sample in which N1 and/or N2 is detected (Ct <40) are positive. Invalid samples were evaluated by RP (Ct > 35).

## Results

A total of 1,120 subjects were enrolled for the study. The median age of enrolled subjects was 35 (Interquartile range, IQR, 27–44), of which 9.9% were females (111) and 90.1% males (1,009). Twelve subjects were excluded from the study; 6 because they entered the prison with a positive COVID-19 diagnosis, and 6 because of an immediate release (same day or the day after) from custody.

### Protocol I

**Figure 1:**
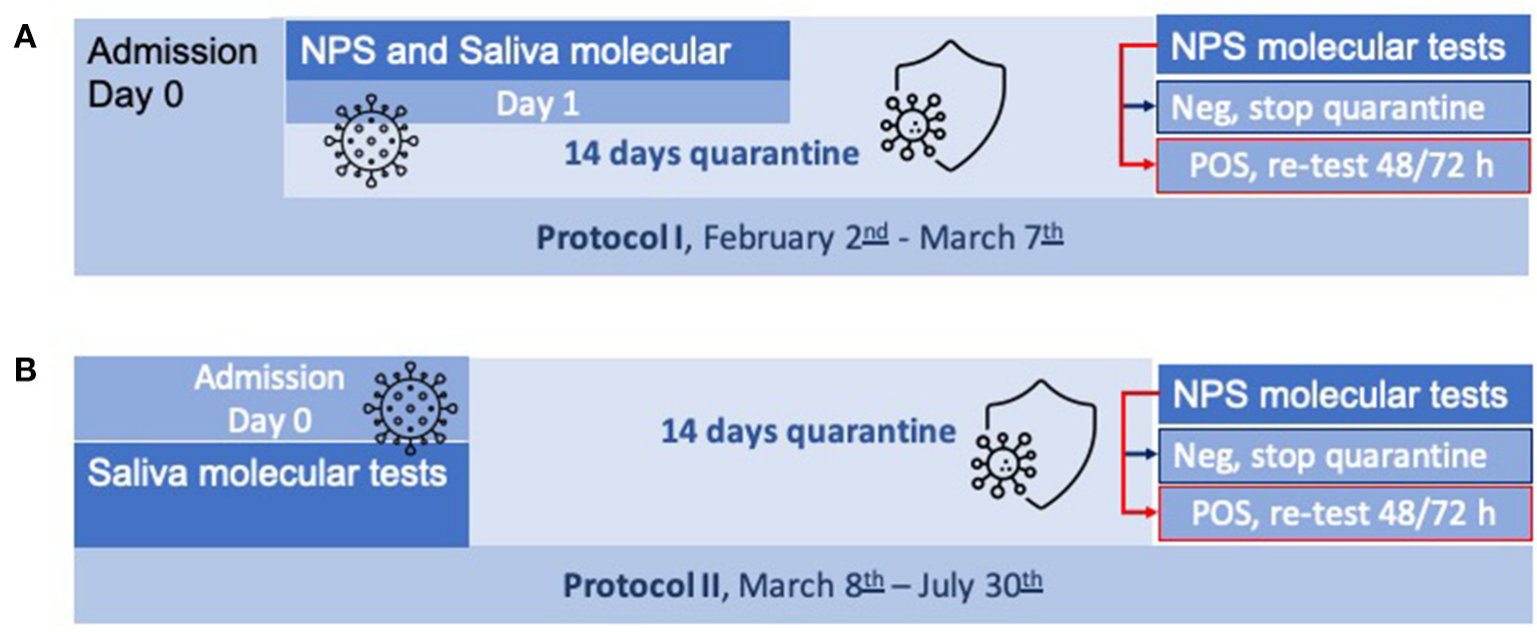
**(A,B)** Workflow of the two applied protocols. NPS, nasopharyngeal swab; Neg, negative; POS, positive.

When prison supervision began, both nasopharyngeal swabs and saliva molecular tests were performed on the first working day after custody (Figure 1A). During this first period, we collected 165 saliva samples and 156 nasopharyngeal swabs, as 9 subjects refused the NPS testing. Among simultaneously collected saliva and NPS, 150 subjects tested negative in both tests, (1) tested weakly positive only in saliva, and 3 were weakly positive in the nasopharynx alone. (2) Subjects tested positive in NPS and negative in saliva, but saliva collection was performed upon food ingestion.

As food and beverage intake before saliva test strongly impacts the test reliability (10), correction measures were introduced to avoid the scarce PiP compliance to saliva collection protocol.

### Protocol II

During this second period (Figure 1B), we collected 943 saliva. Among them, 905 (98.7%) tested negative, one tested positive (0.1%) and it was confirmed by NPS test, and 4 saliva samples tested weakly positive (0.4%), not confirmed by subsequent NPS test. Twenty-eight subjects collected a scarce saliva quantity and were not processed (3%). Three saliva samples displayed high housekeeping (RP) Ct value, and were considered invalid, whereas 2 samples tested positive for only the viral gene N2 with a Ct value above 38. All subjects tested negative at the NPS control test to exit the quarantine.

## Discussion

In response to COVID-19 outbreaks in institutionalized settings, World Health Organization (WHO) guidelines recommend that prison and health agencies engage in prevention and control, risk management, treatment, and information sharing (2). Preventing COVID-19 is particularly urgent for overcrowded prisons because they are linked to transmission of infections and adverse health outcomes (11–13).

Detection of SARS-CoV-2 in asymptomatic carriers is urgently needed for the pandemic containment and prevention of outbreaks. Self-collected saliva should be considered a feasible and cheaper alternative for mass screening of SARS-CoV-2 in asymptomatic persons because it provides highly accurate results (14).

Saliva samples have already been suggested as tools for the detection of respiratory viruses in order to reduce the cost and time associated with collections (14, 15). Studies have shown that the detection of respiratory viruses in salivary samples has high sensitivity and specificity (16, 17). In addition, the collection of saliva samples is less invasive and carried out independently, hence generally more acceptable. Saliva sampling do not require the presence of health workers (nurses and doctors) allowing task shifting, require less training, reduce operator dependent variability and the risk of infection.

In Protocol I, saliva samples were collected in the morning, after breakfast. Many PiP had eaten, leading to less reliable results as reported in the literature (10). Since the introduction of Protocol 2, such bias was avoided. In this setting, out of 156 paired saliva and NPS tests, 4 showed discordant results, probably due to the known difference in infection kinetics in oral cavity vs. respiratory tract (18). The low number of positive subjects reflects the regional viral circulation in the considered period^1^ Nevertheless, it is important to note that applying this protocol led to outbreak avoidance. A possible limit to the saliva use in PiP is the known effect of many recreational drugs on reducing saliva production (19). Such limit can be easily overcome by extending the time of collection to 3 min. On the other hand, performing nasopharyngeal swab in PiP with mental health disorders and/or drug users is much more difficult than the saliva testing, especially at the entry, when they experience the main challenge of acute stress of the incarceration.

The simplicity of saliva sampling showed a higher compliance to the test and a subsequent reduction of refusal to be screened.

Improving compliance to public health measures is fundamental both in the general population and in prisons. Indeed, addressing the known vaccine hesitancy (20) would strongly enhance the success of immunization coverage in this fragile population.

## Conclusions

We believe that this study provides evidence that saliva testing would be an ideal solution to operate in contexts such as the prison setting. Clearly, one of the key factors the COVID-19 pandemic control has been the mass vaccination. However, breakthrough infections from new viral variants are rising, underlying the need to monitor viral circulation especially in closed communities.

## Data availability statement

The raw data supporting the conclusions of this article will be made available by the authors, without undue reservation.

## Ethics statement

Ethical review and approval was not required for the study on human participants in accordance with the local legislation and institutional requirements. Written informed consent for participation was not required for this study in accordance with the national legislation and the institutional requirements.

## Author contributions

CP, EO, NC, RG, RR, and EB conceived the project and analyzed data. CP, EO, SB, SA, and VM performed the experiments. CP, EO, NC, RG, RR, EB, RB, and BP collected and analyzed data. CP, EO, EB, and NC wrote the manuscript. RG, RR, and EB provided guidance in the manuscript revision and data interpretation. All authors contributed to the article and approved the submitted version.

## Conflict of interest

The authors declare that the research was conducted in the absence of any commercial or financial relationships that could be construed as a potential conflict of interest. The reviewer VC declared a past co-authorship/collaboration with the authors RG and RR to the handling Editor.

## Publisher's note

All claims expressed in this article are solely those of the authors and do not necessarily represent those of their affiliated organizations, or those of the publisher, the editors and the reviewers. Any product that may be evaluated in this article, or claim that may be made by its manufacturer, is not guaranteed or endorsed by the publisher.

## Footnotes

^1^ https://www.epicentro.iss.it/coronavirus/SARS-CoV-2-dashboard
